# Mental Health and Survival in Medicare Beneficiaries With Lung and Head and Neck Cancer

**DOI:** 10.1002/pon.70510

**Published:** 2026-06-04

**Authors:** Stephanie Misono, Jesse R. Fann, Nosayaba Osazuwa‐Peters, Schelomo Marmor

**Affiliations:** ^1^ Department of Otolaryngology—Head and Neck Surgery University of Minnesota Minneapolis Minnesota USA; ^2^ Department of Psychiatry and Behavioral Sciences University of Washington School of Medicine Seattle Washington USA; ^3^ Clinical Research Division Fred Hutchinson Cancer Center Seattle Washington USA; ^4^ Department of Head and Neck Surgery & Communication Sciences Duke University School of Medicine Durham North Carolina USA; ^5^ Duke Cancer Institute Durham North Carolina USA; ^6^ Department of Population Health Sciences Duke University School of Medicine Durham North Carolina USA; ^7^ Center for Learning Health Systems Science Clinical Quality Outcomes Discovery and Evaluation Core (CQODE) University of Minnesota Minneapolis Minnesota USA; ^8^ Department of Surgery University of Minnesota Minneapolis Minnesota USA

**Keywords:** distress, medicare, mental health, oncology, survival

## Abstract

**Background:**

Mental health (MH) conditions can significantly influence cancer outcomes, yet associations with survival and potential impacts of MH care utilization remain underexplored.

**Aims:**

Examine associations between (1) MH diagnoses and (2) MH utilization and survival among Medicare beneficiaries with lung or head and neck (HN) cancers.

**Methods:**

Patients diagnosed with lung or HN cancers between 2004 and 2018 were stratified by MH diagnosis status in the two years prior to cancer diagnosis. We examined factors associated with MH diagnosis and MH services and evaluated overall and cancer specific survival analyses using Kaplan‐Meier survival analyses and multivariable Cox regressions.

**Results:**

19% of patients with a lung cancer diagnosis (*n* = 333,875) and 15% of HN patients (*n* = 39,253) received a MH diagnosis within two years before cancer diagnosis. Kaplan‐Meier survival analysis indicated significantly worse survival among patients with a MH diagnosis and higher rates of survival among those receiving MH services. Among individuals with a MH diagnosis, the mortality hazard for individuals that received MH services was lower compared to those who did not receive MH services, in both HN and Lung cancer.

**Conclusions:**

MH conditions were common in people with lung and HN cancers and associated with worse overall and cancer‐specific survival for head and neck cancer. Among those with MH diagnoses, receipt of MH services was associated with lower hazard of death, highlighting the need for early identification of this at‐risk population and integration of MH care into oncology care.

## 1 Introduction

Mental health (MH) problems are common in people with cancer. A substantial proportion of patients with cancer experience emotional and psychological distress (35%–80%), including symptoms of anxiety (10%–40%), and depression (5%–30%) [1, 2, 3, 4, 5]. In multiple cancer types, including lung, colon, head and neck (HN), prostate, bladder, and breast cancers, pre‐existing and co‐existing mental health problems have been associated with worse overall and/or cancer‐specific survival [6, 7, 8, 9, 10, 11]. These findings are not universal across cancer types, however [12]. Head and neck and lung cancers are among the cancer types particularly associated with high distress, as well as post‐cancer sequelae communication disorders, as corroborated by the high incidence of suicide among patients with these cancers [4, 13, 14].

Many cancer patients with mental health conditions do not receive adequate mental health care, despite the potential for identification at the time of cancer diagnosis as well as the significant impact these conditions have on overall well‐being and survival [15, 16]. Prior work examining associations between pre‐existing MH disorders, MH treatment, and survival in lung and head and neck cancers has noted that survivorship care for these patients can be intense and complex, emphasizing the need for integrated mental health and oncology care to improve treatment outcomes. In people with lung cancer diagnosed in 2003–2005, baseline depression symptoms were associated with worse survival, and in a hopeful turn, those with subsequently improved depression ultimately had survival rates similar to those without depression at baseline [8]. In a more recent cohort of people with lung cancer in the US military health system, pre‐existing MH diagnoses were associated with worse overall and cancer‐specific survival, and receiving mental health care was associated with better overall and disease‐specific survival [17]. Similarly, in head and neck cancer, depression before or following the cancer diagnosis was associated with worse overall and cancer‐specific survival [18, 19]; less is known about the impact of MH care on these challenges although some literature suggests it is protective [19].

This study examines the associations between MH diagnoses and utilization with overall and cancer‐specific survival among Medicare beneficiaries with lung or HN cancers. We hypothesized that (1)MH diagnoses would be associated with worse overall survival and cancer‐specific survival than in patients without MH diagnoses, and (2) MH care utilization would have a partially protective effect on survival.

## 2 Methods

### 2.1 Data

We used data from the Surveillance, Epidemiology, and End Results (SEER)‐Medicare‐linked database. SEER‐Medicare combines national SEER cancer registry information to identify cancer cases and link with Medicare enrollment and claims data for patients in the registry covered by Medicare between 2004 and 2017, which was the most recent data release at the time of study initiation. The dataset linkage includes demographics, tumor characteristics, healthcare utilization, diagnoses, procedures, and mortality with follow up through 2018. The SEER‐Medicare Patient Entitlement and Diagnosis Summary File includes patient demographic and tumor characteristics, including age at diagnosis, race, primary tumor site, histology type, tumor stage, vital status, and cause of death (as per death certificate). Claims for hospitalizations and inpatient procedures are available in the Medicare Provider Analysis and Review and National Claims History files, and office visits are captured through a combination of National Claims History files for provider charges and outpatient Standard Analytical Files for facility charges. We used the claims data to calculate Charlson Comorbidity index weights that consider the International Classification of Diseases, Ninth and Tenth Revision (ICD‐9/ICD‐10) diagnosis codes, ICD‐9/ICD‐10 procedure codes, and Healthcare Common Procedure Coding System procedure codes.

### 2.2 Cohort Selection

We included patients diagnosed with lung cancer or head and neck cancer between 2004 and 2017, aged 67 years and older, enrolled in fee‐for‐service (FFS) Medicare Part A and B for at least 24 months before their cancer diagnosis (i.e., no Medicare Advantage enrollment) to allow for identifying mental illness diagnoses during that period. We included patients whose lung cancer or head and neck cancer was their primary malignancy (Table S1). We excluded patients diagnosed posthumously (via autopsy or death certificates).

### 2.3 Mental Health Diagnoses

Pre‐existing mental health conditions were identified using ICD‐9 and ICD‐10 codes based on the methodology from the Chronic Conditions Warehouse (CCW) formulas [20]. (Table S1). These formulas, developed by the Centers for Medicare & Medicaid Services, are validated standard algorithms widely used in the literature to assess comorbidities from administrative claims data [21, 22]. Patients were considered to have a MH diagnosis if they had at least one MH CCW diagnosis code in the 2 years prior to cancer diagnosis [23, 24], which reflects at least 1 inpatient claim OR 2 other non‐drug claims of any service type related to those MH codes [20]. Only pre‐existing MH diagnoses were examined for this study, because from a pragmatic clinical perspective, those individuals could be identified as higher risk at the time of cancer diagnosis from information already present in their records. Consistent with the CMS definitions, we also created a MH services variable that was defined as any MH visit after MH diagnosis [25, 26].

### 2.4 Covariates

Sociodemographic covariates derived from SEER data included age at lung or head and neck cancer diagnosis, marital status, rurality (based on Rural‐Urban Commuting Area Codes [27]). A combined race and ethnicity variable, based on SEER‐reported race and ethnicity, was included. Health‐related variables included the modified Charlson Comorbidity Index (CCI; excluding all cancer diagnoses) [28].

### 2.5 Statistical Analysis

We performed parallel analyses in patients by cancer type (Lung and HN cancer). Participant characteristics by MH status and bivariate associations between cancer stage at diagnosis and independent variables were assessed using chi‐square tests to provide descriptive context and to illustrate baseline differences prior to adjustment. For multivariable analysis, we used logistic regression to account for and reflect the simultaneous contributions and effects of all modeled covariates. The factors associated with MH at diagnosis were modeled, adjusting for age, race and ethnicity, marital status, rural/urban geography, comorbidity score, and stage. Survival was analyzed using both Kaplan‐Meier methods and Cox proportional hazards modeling for each cancer type. Fine‐Gray models [29] were used as part of a sensitivity analysis to account for competing risk of mortality, ensuring that our findings were not an overestimation of risk and not confounded by differential mortality rates. Additionally, we stratified patients by stage to ensure that findings were consistent. All analyses were conducted using SAS 9.4, with a critical alpha of 0.05. Given that this dataset is retrospective and de‐identified this study was deemed exempt from review by the University of Minnesota Institutional Review Board (STUDY00014220).

## 3 Results

### 3.1 Prevalence of Mental Health Diagnoses

We identified a final cohort of 333,875 lung cancer patients and 39,253 HN cancer patients, of whom 19% and 15%, respectively, had at least one MH diagnosis in the two years prior to their cancer diagnosis (Table 1). Among lung cancer patients with MH diagnoses, most were 65–74 years old (56% *n* = 35,343). For HN cancer patients, similar trends were observed, with older adults (age 75+) comprising 43% of the MH‐diagnosed group versus 46% of those without a MH diagnosis. In the lung cancer cohort, females constituted 62% of those with MH diagnoses and 48% of those without, and in the HN cohort, 46% of MH‐diagnosed patients were female, compared to 34% in the non‐MH‐diagnosed group.

**TABLE 1:** Basic characteristics of medicare beneficiaries with head and neck cancer and lung cancer with and without a mental health (MH) diagnosis.

	Lung cancer *N* = 333,875			Head and neck cancer *N* = 39,253		
	No MH diagnosis (*n* = 270,218; 81%)	MH diagnosis (*n* = 63,657; 19%)		No MH diagnosis (*n* = 33,524; 85%)	MH diagnosis (*n* = 5729; 15%)	
	*n* (%)	*n* (%)	aOR 95% CI	*n* (%)	*n* (%)	aOR 95% CI
Age						
67–75	126,078 (47)	35,343 (56)	**REF**	18,042 (54)	3237 (57)	**REF**
75+	144,140 (53)	28,314 (44)	0.65 (0.64–0.66)	15,482 (46)	2492 (43)	0.73 (0.69–0.77)
Sex						
Male	140,967 (52)	24,440 (38)	**REF**	21,978 (66)	3122 (54)	**REF**
Female	129,251 (48)	39,217 (62)	1.74 (1.71–1.77)	11,546 (34)	2607 (46)	1.70 (1.60–1.80)
Marriage status						
Yes	93,783 (35)	18,657 (29)	**REF**	12,452 (37)	1881 (33)	**REF**
No	83,291 (31)	23,212 (36)	1.12 (1.10–1.15)	9836 (29)	1872 (33)	1.09 (1.02–1.18)
Missing	93,144 (34)	21,788 (34)	1.34 (1.31–1.38)	11,236 (34)	1976 (35)	1.10 (1.02–1.19)
Race						
NHW	228,359 (84)	57,783 (91)	**REF**	29,001 (87)	5205 (91)	**REF**
Black	23,708 (9)	4063 (6)	0.65 (0.63–0.67)	2089 (6)	319 (5)	0.95 (0.85–1.06)
Other	18,151 (7)	1811 (3)	0.44 (0.42–0.46)	2434 (7)	205 (4)	0.85 (0.60–1.21)
Urban/Rural						
Urban	248,779 (92)	58,189 (91)	**REF**	30,957 (92)	5293 (92)	**REF**
Rural	19,315 (7)	4872 (8)	1.03 (0.99–1.06)	2325 (7)	399 (7)	0.99 (0.88–1.10)
Missing	2124 (1)	596 (1)	1.13 (1.03–1.24)	242 (1)	37 (1)	0.89 (0.62–1.26)
Stage						
I–II	93,702 (35)	25,191 (40)	**REF**	20,451 (61)	3377 (59)	**REF**
III	9008 (3)	2642 (4)	1.00 (0.95–1.05)	2840 (8)	523 (9)	1.13 (1.01–1.25)
IV	10,1877 (38)	22,367 (35)	0.91 (0.89–0.93)	5362 (16)	1088 (19)	1.32 (1.22–1.42)
Missing	65,631 (24)	13,457 (21)	0.72 (0.70–0.74)	4871 (15)	741 (13)	0.92 (0.84–1.01)
Charlson index						
0	132,887 (49)	24,066 (38)	**REF**	21,731 (65)	2799 (49)	**REF**
1	53,198 (20)	14,964 (23)	1.52 (1.48–1.55)	5394 (16)	1166 (20)	1.68 (1.56–1.82)
2+	84,133 (31)	24,627 (39)	1.69 (1.66–1.73)	6399 (19)	1764 (31)	2.28 (2.13–2.43)

*Note:* All bolded values are significant to the *p* ≤ 0.05.

### 3.2 Factors Associated With MH Diagnoses

In multivariable models (Table 1), older age was inversely associated with MH diagnoses in both cancer types. For lung cancer patients aged 75+, the odds of an MH diagnosis were 0.65 times lower (95% Confidence Interval (CI): 0.64–0.66) compared to those aged 67–75 years old. In HN cancer, similar patterns emerged, with an odds ratio (OR) of 0.73 (95% CI: 0.69–0.77) for the older age group. Sex and marital status also influenced MH diagnosis likelihood. Females with lung cancer were 1.74 times more likely to have an MH diagnosis than males (95% CI: 1.71–1.77). In HN cancer, the corresponding OR was 1.70 (95% CI: 1.60–1.80). Married individuals in both cohorts were less likely to have an MH diagnosis compared to their unmarried counterparts. Individuals with higher Charlson comorbidity index (CCI) scores were more likely to have a MH diagnosis in both lung 1.69 (95% CI: 1.66–1.73) and HN 2.28 (95% CI: 2.13–2.43).

### 3.3 MH Diagnoses, Utilization, and Survival

**FIGURE 1:**
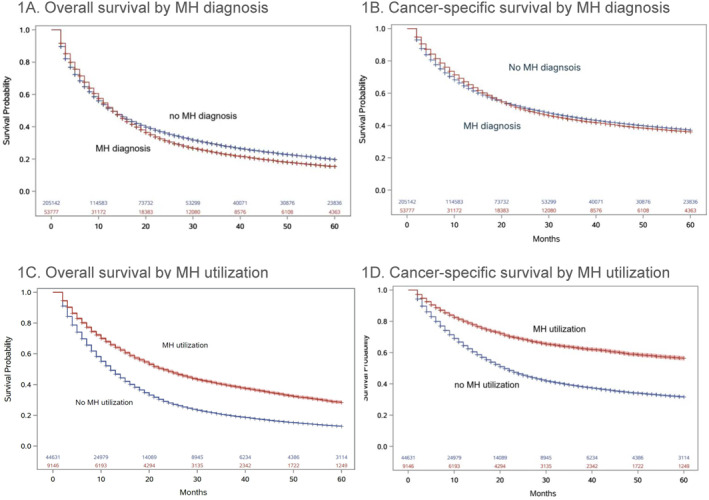
Kaplan‐Meier curves in patients with lung cancer.

In the lung cancer cohort, unadjusted Kaplan‐Meier mortality estimates were similar between patients who did (13 months, [95% CI: 13–14]) and did not (13 months, [95% CI: 12–14]) receive a MH diagnosis (Figure 1A). Among the lung cancer patients with a MH diagnosis, 16% had MH utilization. The median overall survival among those who had MH utilization was 19 months (95% CI: 18–20), whereas the median survival among those who did not utilize MH services was 9 months (95% CI: 8–10) (Figure 1C). Findings were similar for cancer‐specific survival (Figure 1B, 1D).

**FIGURE 2:**
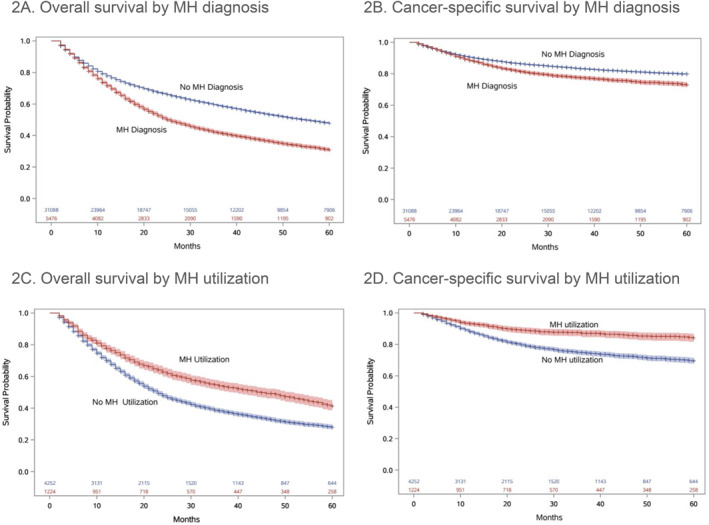
Kaplan‐Meier curves in patients with HN cancer.

In the HN cohort, unadjusted Kaplan‐Meier mortality estimates demonstrated significantly lower survival for patients who received a MH diagnosis compared with those who did not (Figure 2A), with median survival 55 months [95% CI: 53–56] for no MH diagnosis versus 25 months [95% CI: 24–27] for MH diagnosis (HN). Among the HN cancer patients with a MH diagnosis, 22% had MH utilization. The median overall survival among those who had MH utilization was 23 months [95% CI: 22–24], and among those who did not utilize MH services was 12 months [95% CI: 12–13] (Figure 2C). Findings were similar, though attenuated somewhat, for cancer‐specific survival (Figure 2B, 2D).

In adjusted Cox models the overall Hazard of Death was higher for individuals with HN cancer and MH diagnosis (Hazard Ratio (HR) 1.40, 95% CI 1.35–1.46) but not for those with Lung cancer and MH diagnosis (HR 0.99, 95% CI 0.98–1.01) (Table 2). Among individuals with a MH diagnosis, the Hazard of Death associated with receipt of MH services was lower for both patients with HN (HR 0.64, 95% CI 0.58–0.70) and patients with Lung (HR 0.65, 95% CI 0.63–0.66) cancers when adjusting for all other factors (Table 3). Similar to overall survival, cancer‐specific survival was also better among patients with Lung and HN cancers who received MH services (Table S2).

**TABLE 2:** Cox model and overall hazard of death among lung (model 1) and head and neck (model 2) cancers with a mental health diagnosis.

	Lung	HN
	Hazard ratio (95% CI)	Hazard ratio (95% CI)
Age		
67–75	1.00 Referent	1.00 Referent
75+	1.33 (1.32–1.34)	1.53 (1.49–1.58)
Sex		
Male	1.00 Referent	1.00 Referent
Female	0.82 (0.82–0.83)	0.88 (0.85–0.91)
Marriage status		
Yes	1.00 Referent	1.00 Referent
No	1.22 (1.20–1.23)	1.42 (1.37–1.47)
Missing	0.81 (0.80–0.82)	0.92 (0.88–0.96)
Race		
NHW	1.00 Referent	1.00 Referent
Black	1.02 (1.02–1.03)	1.38 (1.30–1.46)
Other	0.82 (0.81–0.83)	0.87 (0.81–0.93)
Urban/Rural		
Urban	1.00 Referent	1.00 Referent
Rural	1.12 (1.10–1.13)	1.03 (0.97–1.09)
Missing	1.16 (1.11–1.20)	0.99 (0.83–1.19)
Stage		
I–II	1.00 Referent	1.00 Referent
III	1.63 (1.59–1.67)	1.34 (1.25–1.43)
IV	3.22 (3.19–3.25)	2.29 (2.20–2.28)
Missing	3.72 (3.68–3.76)	2.12 (2.03–2.21)
Charlson index		
0	1.00 Referent	1.00 Referent
1	1.02 (1.00–1.03)	1.21 (1.16–1.26)
2+	1.18 (1.17–1.19)	1.75 (1.69–1.26)
Mental health diagnosis		
No	1.00 Referent	1.00 Referent
Yes	0.99 (0.98–1.01)	1.40 (1.35–1.46)

**TABLE 3:** Cox model and overall survival hazard of death among lung (model 1) and head and neck (HN) (model 2) cancers with a mental health diagnosis and with and without mental health service.

	Lung	HN
	Hazard ratio (95% CI)	Hazard ratio (95% CI)
Age		
67–75	1.00 Referent	1.00 Referent
75+	1.21 (1.19–1.23)	1.36 (1.49–1.58)
Sex		
Male	1.00 Referent	1.00 Referent
Female	0.80 (0.78–0.81)	0.80 (0.75–0.86)
Marriage status		
Yes	1.00 Referent	1.00 Referent
No	1.15 (1.12–1.18)	1.22 (1.12–1.32)
Missing	0.84 (0.82–0.87)	0.86 (0.78–0.94)
Race		
NHW	1.00 Referent	1.00 Referent
Black	1.10 (1.06–1.14)	1.32 (1.15–1.51)
Other	0.88 (0.84–0.94)	0.94 (0.77–1.14)
Urban/Rural		
Urban	1.00 Referent	1.00 Referent
Rural	1.07 (1.03–1.11)	0.96 (0.84–1.09)
Missing	1.04 (0.95–1.15)	0.99 (0.66–1.49)
Stage		
I–II	1.00 Referent	1.00 Referent
III	1.65 (1.57–1.74)	1.26 (1.09–1.45)
IV	2.88 (2.82–2.95)	1.77 (1.63–1.93)
Missing	2.71 (2.63–2.79)	1.77 (1.59–1.96)
Charlson index		
0	1.00 Referent	1.00 Referent
1	1.03 (1.01–1.06)	1.23 (1.12–1.35)
2+	1.14 (1.11–1.16)	1.63 (1.50–1.76)
Mental health services		
No	1.00 Referent	1.00 Referent
Yes	0.65 (0.63–0.66)	0.64 (0.58–0.70)

## 4 Discussion

Findings from this large cohort of 373,128 U.S. adults with lung or HN cancer and a MH diagnosis provide valuable insights into the relationship between MH and cancer outcomes. Patients with HN cancer and preexisting MH diagnoses had lower overall survival and cancer‐specific survival compared to those without MH diagnoses. While no significant differences in overall survival were observed among lung cancer patients with or without preexisting MH diagnoses, cancer‐specific survival was lower among those with MH diagnoses. In both cancer types, MH care utilization among individuals with preexisting MH diagnoses was associated with a partially protective effect on survival outcomes, underscoring the critical importance of screening for a history of MH problems and integrating MH care into oncology practice.

In this study 19% and 15%, respectively, had a mental health (MH) diagnosis within 2 years prior to cancer diagnosis, consistent with prior research highlighting the high prevalence of MH disorders in cancer patients. Our observed prevalence of MH diagnoses in patients with lung cancer was lower than previously reported [17], whereas our observed prevalence in HN cancer patients was similar to the depression rates reported in MarketScan and SEER‐Medicare studies [30, 31].

Our findings add to the literature regarding the association of MH diagnoses and survival outcomes in cancer. A systematic review examining the impact of pre‐existing mental health disorders on guideline‐recommended cancer treatment, treatment delays, and compliance [32] showed that individuals with pre‐existing mental health disorders were less likely to be allocated to guideline‐recommended cancer treatment for breast, prostate, colon, rectal, bladder, pancreatic, esophageal, head/neck, and lung cancer [32]. In lung cancer, military veterans (VA) with a MH disorder had an 11% higher mortality risk compared to those without [17]. The contrast between our findings and the prior study may be related to VA and Medicare FFS differences. In a smaller study, depression was independently associated with poor cancer treatment adherence and worse prognosis in advanced non‐small cell lung cancer [33]. In our study, among patients with lung cancer, overall and cancer‐specific survival were essentially the same regardless of the presence of MH diagnosis. This may be in part a reflection of early and high cancer‐related mortality in people with lung cancer, which may obscure detection of a potential effect of MH on survival. The association of mental health conditions and decreased survival may also be influenced by unhealthy behaviors such as tobacco and alcohol use and sedentary lifestyle, and unhealthy diet [34, 35].

In HN cancer, our study demonstrated that preexisting MH diagnoses were associated with a significantly higher risk of all‐cause and cancer‐specific mortality, consistent with prior research [36]. Similarly, a systematic review found that pretreatment depression or depressive symptoms were associated with worse overall survival (OS) in HN patients, with a pooled hazard ratio (HR) of 1.33 (95% CI, 1.16–1.52; *p* < 0.0001) [6]. Our findings for HN cancers HR 1.30 (1.23–1.37) are comparable to these previously reported effect sizes, underscoring the adverse impact of MH conditions on survival outcomes in this population.

Prior studies have reported that MH utilization via psychotherapy and/or medications such as antidepressants can be associated with better survival in patients with cancer and mental health concerns [37], but the literature on the impact of MH utilization specifically in individuals with *preexisting* MH conditions is more limited, and interactions between psychotherapy and medications can be complex and difficult to assess. In lung cancer, a VA cancer registry study demonstrated that in veterans with preexisting mental health disorders and non‐small cell lung cancer, participation in mental health treatment programs and social support services was linked to improved cancer outcomes, including reduced mortality [38]. Our findings are more broadly representative of health care utilization in the general and Medicare FFS population, although our study focused on non‐medication mental health care utilization. In HN cancer, while there is a significant burden of mental health concerns, there is a paucity of data on mental health utilization, even though utilization is associated with better overall survival for patients. A recent study from the Netherlands examining 610 head and neck cancer patients from diagnosis to two years post‐treatment reported that the rate of mental healthcare utilization, including psychology, psychiatry, and psychotherapist visits, in patients with HN cancer was generally low (5%–9%) but higher among those with psychological symptoms, mental disorders, or unmet care needs. Utilization was significantly associated with anxiety symptoms, care needs, younger age, advanced disease stage, lower self‐efficacy, and greater social support seeking [39].

Establishing resources for psychosocial oncology services has been reinforced by the Oncology Care Model (OCM), introduced by the Center for Medicare & Medicaid Services, which mandates the provision of psychosocial support [40]. However, access to mental health services remains challenging, particularly for patients on Medicare, where mental health care options are often insufficient to meet the needs of cancer patients [41]. Systemic gaps in mental health care availability further exacerbate disparities in cancer outcomes, emphasizing the urgent need for evidence‐based integrated mental health services within routine oncology care, such as via the collaborative care model [42, 43]. Leveraging telehealth, group treatment, incorporating innovative care approaches, such as web‐based and mobile interventions, and guidelines for MH resource availability may also help to address this critical need. Concerted efforts are imperative to expand and enhance mental health services for patients with cancer, with a steadfast commitment to ensuring their psychosocial well‐being.

### 4.1 Study Limitations

Despite the important findings of this study, several limitations should be noted. Our study cohort of Medicare beneficiaries aged 67 years and older limits the generalizability of our findings to younger populations, or those enrolled in other insurance programs. In addition, medication and medication‐management visits were not a focus of this analysis because the use of psychoactive medications in the setting of acute cancer treatment and/or associated hospitalizations would be difficult to interpret. Results could also be affected by the specific time windows for mental health diagnoses that were used in our study, although these were selected according to current best practice. Our reliance on Medicare claims data ensured that all covered services were captured. However, this dataset does not measure adherence to mental health care or capture mental health services received outside of Medicare FFS, such as self‐paid or out‐of‐network care. In addition, future data releases may facilitate examination of mental health utilization in the evolving landscape of health care access over time.

Treating MH as a binary variable is a simplification that may reflect under‐detection of true MH problems [44], and also could obscure the potential differential contributions of different subgroups such as those with serious mental illness (SMI) such as schizophrenia and major depressive disorder. In this study, we conducted post‐hoc sensitivity analyses comparing survival among those with SMI to those with other MH diagnoses and those with no MH diagnoses [45]. The survival differences were observed for both SMI and other MH groups as compared to the no MH diagnosis group, suggesting that the observed findings were not attributable exclusively to the presence of SMI. Nonetheless, moving beyond a binary definition for MH is likely to add valuable nuance to future studies.

As with any study of this type, unmeasured confounding is another potential limitation. For example, individuals with undiagnosed or untreated mental illness may differ systematically from those with diagnosed mental illness or those without mental illness altogether, such as in care‐seeking behavior, social support, access to integrated care systems, overall health care utilization and/or medication use. Related topics such as mental health diagnoses following cancer diagnosis (as opposed to preceding it) and substance use/abuse were not a focus of this study but could be an important area for future studies. Finally, our study design does not allow for causal inferences, and it is possible that other factors, such as healthcare access or mental health provider practice patterns and availability, influenced both the diagnosis of mental illness and cancer outcomes.

### 4.2 Clinical Implications

Future research should investigate the mechanisms by which mental health care contributes to improved survival, including its impact on treatment adherence, symptom management, health behaviors, and overall quality of life. Additionally, health systems should implement strategies to enhance access to evidence‐based mental health care for cancer patients, such as using the collaborative care model and leveraging remote mental health interventions to reach underserved populations. Addressing mental health in oncology settings remains a critical component of comprehensive cancer care. By prioritizing mental health interventions, health systems can improve both psychological well‐being and survival outcomes for patients facing the dual burden of cancer and mental health disorders.

## 5 Conclusion

In this study, we examined the relationship between mental health diagnoses, mental health service utilization, and survival outcomes among Medicare beneficiaries with lung or HN cancers. Preexisting mental health diagnoses were prevalent among individuals with lung and HN cancers, and preexisting mental health conditions were associated with lower overall and cancer‐specific survival among patients with HN cancer, but less so in lung cancer. Importantly, among individuals with preexisting mental health diagnoses, receiving mental health services was associated with substantially better survival in both cancer types. These findings suggest that access to and engagement with mental health care could mitigate some of the adverse effects associated with mental health disorders in cancer patients, highlighting an important priority area for integrated oncology care.

## Author Contributions

S.M. conceptualized and designed the study, led data acquisition, analysis, and interpretation, drafted the manuscript, revised and approved the final version. J.R.F. provided content expertise in psycho‐oncology, contributed to interpretation of findings, reviewed and edited manuscript for intellectual content, approved the final version. N.O.P. provided content expertise in psycho‐oncology, contributed to interpretation of findings, reviewed and edited manuscript for intellectual content, approved the final version. S.M. conceptualized and designed the study, led data acquisition, analysis, and interpretation, drafted the manuscript, revised and approved the final version.

## Funding

The authors have nothing to report.

## Conflicts of Interest

The authors declare no conflicts of interest.

## Supporting information


**Table S1:** Codes for lung and head and neck (HN) cancer, mental health (MH) diagnosis, and MH utilization.

**Table S2:** Cox model and cancer specific survival hazard of death among lung (model 1) and head and neck (HN) (model 2) cancers with a mental health diagnosis and with and without mental health service.

## Data Availability

The data that support the findings of this study are openly available in SEER‐MEDICARE at https://healthcaredelivery.cancer.gov/seermedicare/.

## References

[1] L. E. Carlson , E. L. Zelinski , K. I. Toivonen , et al., “Prevalence of Psychosocial Distress in Cancer Patients Across 55 North American Cancer Centers,” Journal of Psychosocial Oncology 37, no. 1 (January 2019): 5–21, PMID: 30592249, 10.1080/07347332.2018.1521490. 30592249

[2] A. Mehnert , E. Brähler , H. Faller , et al., “Four‐Week Prevalence of Mental Disorders in Patients With Cancer Across Major Tumor Entities,” Journal of Clinical Oncology 32, no. 31 (November 2014): 3540–3546, PMID: 25287821, 10.1200/jco.2014.56.0086. 25287821

[3] U. Goerling , A. Hinz , U. Koch‐Gromus , J. M. Hufeld , P. Esser , and A. Mehnert‐Theuerkauf , “Prevalence and Severity of Anxiety in Cancer Patients: Results From a Multi‐Center Cohort Study in Germany,” Journal of Cancer Research and Clinical Oncology 149, no. 9 (August 2023): 6371–6379, PMCID: PMC10356888, 10.1007/s00432-023-04600-w. 36757620 PMC10356888

[4] M. C. White , C. Corbett , T. Y. Cannon , T. L. Watts , R. Jiang , and N. Osazuwa‐Peters , “Patient‐Reported Distress in Individuals With Head and Neck Cancer,” JAMA Otolaryngology–Head & Neck Surgery 151, no. 2 (February 2025): 160–169, PMCID: PMC11826365, 10.1001/jamaoto.2024.4357. 39699883 PMC11826365

[5] D. Brandenbarg , S. W. M. C. Maass , O. P. Geerse , et al., “A Systematic Review on the Prevalence of Symptoms of Depression, Anxiety and Distress in Long‐Term Cancer Survivors: Implications for Primary Care,” European Journal of Cancer Care 28, no. 3 (May 2019): e13086, PMCID: PMC9286037, 10.1111/ecc.13086. 31087398 PMC9286037

[6] S. Van der Elst , Y. Bardash , M. Wotman , D. Kraus , and T. Tham , “The Prognostic Impact of Depression or Depressive Symptoms on Patients With Head and Neck Cancer: A Systematic Review and Meta‐Analysis,” Head & Neck 43, no. 11 (November 2021): 3608–3617, PMID: 34525238, 10.1002/hed.26868. 34525238

[7] J. M. Hyer , E. P. Kelly , A. Z. Paredes , D. I. Tsilimigras , A. Diaz , and T. M. Pawlik , “Mental Illness Is Associated With Increased Risk of Suicidal Ideation Among Cancer Surgical Patients,” Americas Journal of Surgery 222, no. 1 (July 2021): 126–132, PMID: 33268055, 10.1016/j.amjsurg.2020.10.028. 33268055

[8] D. R. Sullivan , C. W. Forsberg , L. Ganzini , et al., “Longitudinal Changes in Depression Symptoms and Survival Among Patients With Lung Cancer: A National Cohort Assessment,” Journal of Clinical Oncology 34, no. 33 (November 2016): 3984–3991, PMCID: PMC5477833, 10.1200/jco.2016.66.8459. 27996350 PMC5477833

[9] J. Baillargeon , Y. F. Kuo , Y. L. Lin , M. A. Raji , A. Singh , and J. S. Goodwin , “Effect of Mental Disorders on Diagnosis, Treatment, and Survival of Older Adults With Colon Cancer,” Journal of the American Geriatrics Society 59, no. 7 (July 2011): 1268–1273, PMCID: PMC4006964, 10.1111/j.1532-5415.2011.03481.x. 21732924 PMC4006964

[10] N. J. Sathianathen , Y. Fan , S. L. Jarosek , et al., “Disparities in Bladder Cancer Treatment and Survival Amongst Elderly Patients With a Pre‐Existing Mental Illness,” European Urology Focus 6, no. 6 (November 2020): 1180–1187, PMID: 30797737, 10.1016/j.euf.2019.02.007. 30797737

[11] K. Iglay , M. L. Santorelli , K. M. Hirshfield , et al., “Impact of Preexisting Mental Illness on All‐Cause and Breast Cancer‐specific Mortality in Elderly Patients With Breast Cancer,” Journal of Clinical Oncology 35, no. 36 (December 2017): 4012–4018, PMID: 28934000, 10.1200/jco.2017.73.4947. 28934000

[12] A. Alobaidi , N. A. Nabulsi , B. Talon , et al., “Depressive Symptoms, Mental health‐Related Quality of Life, and Survival Among Older Patients With Multiple Myeloma,” Supportive Care in Cancer 28, no. 9 (September 2020): 4097–4106, PMCID: PMC7308221, 10.1007/s00520-019-05246-6. 31872292 PMC7308221

[13] S. Misono , N. S. Weiss , J. R. Fann , M. Redman , and B. Yueh , “Incidence of Suicide in Persons With Cancer,” Journal of Clinical Oncology 26, no. 29 (October 2008): 4731–4738, PMCID: PMC2653137, 10.1200/jco.2007.13.8941. 18695257 PMC2653137

[14] N. Osazuwa‐Peters , M. C. Simpson , L. Zhao , et al., “Suicide Risk Among Cancer Survivors: Head and Neck Versus Other Cancers,” Cancer 124, no. 20 (October 2018): 4072–4079, PMID: 30335190, 10.1002/cncr.31675. 30335190

[15] A. Y. Naser , A. N. Hameed , N. Mustafa , et al., “Depression and Anxiety in Patients With Cancer: A Cross‐Sectional Study,” Frontiers in Psychology 12 (April 2021): 585534, PMCID: PMC8081978, 10.3389/fpsyg.2021.585534. 33935849 PMC8081978

[16] J. Mwobobia , M. C. White , O. L. Osazuwa‐Peters , et al., “Depression, Non‐Medical Pain Prescriptions, and Suicidal Behavior in Cancer Survivors,” Journal of Cancer Survivorship (January 2025): PMCID: PMC12217687, 10.1007/s11764-024-01740-x. PMC12217687 39821751

[17] J. Lin , K. A. McGlynn , C. A. Carter , et al., “The Impact of Preexisting Mental Health Disorders on the Diagnosis, Treatment, and Survival Among Lung Cancer Patients in the U.S. Military Health System,” Cancer Epidemiology, Biomarkers & Prevention 25, no. 12 (December 2016): 1564–1571, PMCID: PMC5777503, 10.1158/1055-9965.epi-16-0316. PMC5777503 27566418

[18] B. Barber , J. Dergousoff , L. Slater , et al., “Depression and Survival in Patients With Head and Neck Cancer: A Systematic Review,” JAMA Otolaryngology–Head & Neck Surgery 142, no. 3 (March 2016): 284–288, PMID: 26796781, 10.1001/jamaoto.2015.3171. 26796781

[19] R. W. Huang , K. P. Chang , F. Marchi , et al., “The Impact of Depression on Survival of Head and Neck Cancer Patients: A Population‐Based Cohort Study,” Frontiers Oncology 12 (August 2022): 871915, PMCID: PMC9453493, 10.3389/fonc.2022.871915. PMC9453493 36091181

[20] Chronic Conditions Data Warehouse [Internet] . Chronic Conditions Data Warehouse: [cited 2025 Jun 12], https://www2.ccwdata.org/web/guest/condition‐categories‐other.

[21] K. Elliott , E. Haworth , I. Bolnykh , et al., “Breast Cancer Patients With a Pre‐Existing Mental Illness Are less Likely to Receive guideline‐recommended Cancer Treatment: A Systematic Review and meta‐analysis,” Breast 79, no. 103855 (February 2025): 103855, PMCID: PMC11730251, 10.1016/j.breast.2024.103855. 39708443 PMC11730251

[22] J. Mallet , C. Dubertret , and O. Huillard , “Clinical Diagnosis of Mental Disorders Before Cancer Diagnosis,” JAMA Oncology 3, no. 4 (April 2017): 565–566, PMID: 27918761, 10.1001/jamaoncol.2016.5293. 27918761

[23] Chronic Conditions Data Warehouse [Internet] . Chronic Conditions Data Warehouse: [cited 2026 Apr 23], https://www2.ccwdata.org/web/guest/condition‐categories‐other.

[24] C. M. Spettell , T. C. Wall , J. Allison , et al., “Identifying Physician‐Recognized Depression From Administrative Data: Consequences for Quality Measurement,” Health Services Research 38, no. 4 (August 2003): 1081–1102, PMCID: PMC1360934, 10.1111/1475-6773.00164. 12968818 PMC1360934

[25] C. M. Jones , C. Shoff , C. Blanco , J. L. Losby , S. M. Ling , and W. M. Compton , “Overdose, Behavioral Health Services, and Medications for Opioid Use Disorder After a Nonfatal Overdose,” JAMA Internal Medicine 184, no. 8 (August 2024): 954–962, PMCID: PMC11184500, 10.1001/jamainternmed.2024.1733. 38884975 PMC11184500

[26] Medicare & Mental Health Coverage [Internet]. [cited 2025 Jul 8], https://www.cms.gov/files/document/mln1986542‐medicare‐mental‐health‐coverage.pdf.

[27] E. A. Dobis and A. Sanders , Rural‐Urban Commuting Area Codes [Internet]: [cited 2025 Jun 12], https://www.ers.usda.gov/data‐products/rural‐urban‐commuting‐area‐codes.

[28] SEER‐Medicare: Comorbidity SAS Macros [Internet]. [cited 2025 Jun 12], https://healthcaredelivery.cancer.gov/seermedicare/considerations/calculation.html.

[29] J. P. Fine and R. J. Gray , “A Proportional Hazards Model for the Subdistribution of a Competing Risk,” Journal of the American Statistical Association 94, no. 446 (June 1999): 496–509, 10.1080/01621459.1999.10474144.

[30] K. Rieke , E. Boilesen , W. Lydiatt , K. K. Schmid , J. Houfek , and S. Watanabe‐Galloway , “Population‐Based Retrospective Study to Investigate Preexisting and New Depression Diagnosis Among Head and Neck Cancer Patients,” Cancer Epidemiology 43 (August 2016): 42–48, PMID: 27391545, 10.1016/j.canep.2016.06.008. 27391545

[31] J. H. Lee , D. Ba , G. Liu , D. Leslie , B. E. Zacharia , and N. Goyal , “Association of Head and Neck Cancer With Mental Health Disorders in a Large Insurance Claims Database,” JAMA Otolaryngology–Head & Neck Surgery 145, no. 4 (April 2019): 339–344, PMCID: PMC6481424, 10.1001/jamaoto.2018.4512. 30816930 PMC6481424

[32] Y. H. Wang , A. Aggarwal , R. Stewart , and E. A. Davies , “Impact of Pre‐existing Mental Health Disorders on the Receipt of Guideline Recommended Cancer Treatments: A Systematic Review,” Psycho‐Oncology 32, no. 3 (March 2023): 307–330, PMID: 36588188, 10.1002/pon.6081. 36588188

[33] O. Arrieta , L. P. Angulo , C. Núñez‐Valencia , et al., “Association of Depression and Anxiety on Quality of Life, Treatment Adherence, and Prognosis in Patients With Advanced Non‐Small Cell Lung Cancer,” Annals of Surgical Oncology 20, no. 6 (June 2013): 1941–1948, PMID: 23263699, 10.1245/s10434-012-2793-5. 23263699

[34] J. J. Prochaska , S. Das , and K. C. Young‐Wolff , “Smoking, Mental Illness, and Public Health,” Annual Review of Public Health 38, no. 1 (March 2017): 165–185, PMCID: PMC5788573, 10.1146/annurev-publhealth-031816-044618. PMC5788573 27992725

[35] E. R. Jun , S. H. Kim , Y. J. Cho , Y. A. Kim , and J. Y. Lee , “The Influence of Negative Mental Health on the Health Behavior and the Mortality Risk: Analysis of Korean Longitudinal Study of Aging from 2006 to 2014,” Korean Journal of Family Medicine 40, no. 5 (September 2019): 297–306, PMCID: PMC6768842, 10.4082/kjfm.18.0068. 31505911 PMC6768842

[36] K. Rieke , K. K. Schmid , W. Lydiatt , J. Houfek , E. Boilesen , and S. Watanabe‐Galloway , “Depression and Survival in Head and Neck Cancer Patients,” Oral Oncology 65 (February 2017): 76–82, PMID: 28109472, 10.1016/j.oraloncology.2016.12.014. 28109472 PMC8201663

[37] S. A. Lee , C. M. Nam , Y. H. Kim , T. H. Kim , S. I. Jang , and E. C. Park , “Impact of Onset of Psychiatric Disorders and Psychiatric Treatment on Mortality Among Patients With Cancer,” Oncologist 25, no. 4 (April 2020): e733–e742, PMCID: PMC7160313, 10.1634/theoncologist.2019-0396. 31899576 PMC7160313

[38] J. E. Berchuck , C. S. Meyer , N. Zhang , et al., “Association of Mental Health Treatment With Outcomes for US Veterans Diagnosed With Non‐Small Cell Lung Cancer,” JAMA Oncology 6, no. 7 (July 2020): 1055–1062, PMCID: PMC7273310, 10.1001/jamaoncol.2020.1466. 32496507 PMC7273310

[39] F. Jansen , B. I. Lissenberg‐Witte , J. A. Hardillo , et al., “Mental Healthcare Utilization Among Head and Neck Cancer Patients: A Longitudinal Cohort Study,” Psycho‐Oncology 33, no. 1 (January 2024): e6251, PMID: 37955598, 10.1002/pon.6251. 37955598

[40] Oncology Care Model [Internet]. [cited 2025 Jul 8], https://www.cms.gov/priorities/innovation/innovation‐models/oncology‐care.

[41] L. Lin , H. Lin , R. Zhou , B. Liu , K. Liu , and R. Jiang , “Surviving and Thriving: Assessing Quality of Life and Psychosocial Interventions in Mental Health of Head and Neck Cancer Patients,” Asian Journal of Surgery 28 (November 2024): PMID: 39613637, 10.1016/j.asjsur.2024.11.048. 39613637

[42] M. Li , E. B. Kennedy , N. Byrne , et al., “Systematic Review and Meta‐Analysis of Collaborative Care Interventions for Depression in Patients With Cancer,” Psycho‐Oncology 26, no. 5 (May 2017): 573–587, PMID: 27643388, 10.1002/pon.4286. 27643388

[43] P. A. Tsao , J. R. Fann , A. L. Nevedal , L. E. Bloor , S. L. Krein , and M. E. V. Caram , “A Positive Distress Screen…Now What? An Updated Call for Integrated Psychosocial Care,” Journal of Clinical Oncology 41, no. 31 (November 2023): 4837–4841, PMCID: PMC10617941, 10.1200/jco.22.02719. 37441747 PMC10617941

[44] S. Hwang , R. Jayadevappa , J. Zee , et al., “Concordance Between Clinical Diagnosis and Medicare Claims of Depression Among Older Primary Care Patients,” American Journal of Geriatric Psychiatry 23, no. 7 (July 2015): 726–734, PMCID: PMC4634645, 10.1016/j.jagp.2014.08.009. PMC4634645 25256215

[45] J. F. Figueroa , J. Phelan , E. J. Orav , V. Patel , and A. K. Jha , “Association of Mental Health Disorders With Health Care Spending in the Medicare Population,” JAMA Network 3, no. 3 (March 2020): e201210, PMCID: PMC7082719, 10.1001/jamanetworkopen.2020.1210. PMC7082719 32191329

