# Supplementary material for: Mental Health and Survival in Medicare Beneficiaries With Lung and Head and Neck Cancer
Source: Psychooncology. 2026 Jun 4;35(6):e70510. doi: 10.1002/pon.70510 (PMC13238330; doi:10.1002/pon.70510)
Supplement: Supplementary file 1 — Table S1: Codes for lung and head and neck (HN) cancer, mental health (MH) diagnosis, and MH utilization. [file PON-35-e70510-s001.docx]

Supplemental Table 1. Codes for lung and head and neck (HN) cancer, mental health (MH) diagnosis, and MH utilization.

| **Category** | **SEER-Primary site codes** | **HCPCS** | **ICD-10/09** |
| --- | --- | --- | --- |
| Lung cancer | C340-C343, C348-C349 |  |  |
| HN cancer | C000–C009, C019–C029, C030–C039, C040–C049, C050–C069, C079–C089  C090–C091, C092–C109, C110–C119, C129–C140, C142, C148 |  |  |
| MH diagnosis |  |  |  |
| Anxiety Disorders |  |  | F40.00–F40.02, F40.10–F40.11, F40.210–F40.298, F40.8–F40.9, F41.0–F41.1, F41.3, F41.8–F41.9, F42.2–F42.4, F42.8–F42.9, 293.84, 300.00-300.02, 300.09-300.10, 300.20-300.23, 300.29, 300.3, 300.5, 300.89, 300.9 |
| Acute Stress & PTSD |  |  | F45.8, F48.8-F48.9, F93.8, F99 |
| Obsessive-Compulsive & Somatic Disorders |  |  | F42.2-F42.4, F42, R45.2, R45.5-R45.7, F42.2-F42.4, F42.8-F42.9 |
| Psychosocial Symptoms |  |  | R45.2, R45.5–R45.7 |
| Adjustment & Conduct Disorders (ICD-09 codes) |  |  | 308.0-308.4, 308.9, 309.81, 313.0-313.1, 3132.1-3132.2, 313.3, 313.82 |
| Mood Disorders - Depression & Bipolar |  |  | 296.00-296.16, 296.40-296.46, 296.50-296.56, 296.60-296.66, 296.7, 296.80-296.82, 296.89-296.90, 296.99, F30.10-F30.13, F30.2-F30.4, F30.8-F30.9, F31.0, F31.10-F31.13, F31.2, F31.30-F31.32, F31.4-F31.5, F34.81, F31.60-F31.64, F31.70-F31.78, F31.81, F31.89, F319, F338, F34.81, F34.89, F34.9, F39  F30.10–F30.13, F30.2–F30.4, F30.8–F30.9, F31.0, F31.10–F31.13, F31.2, F31.30–F31.32, F31.4–F31.5, F31.60–F31.64, F31.70–F31.78, F31.81, F31.89, F31.9, F33.8 |
|  |  |  |  |
| Major Depressive Disorders |  |  | F06.0, F06.2, F28-F29, F32.3, F33.3, F44.89, F32.A, F33, 296.20-296.26, 296.30-296.36, 3004, 311 |
| Schizophrenia & Psychotic Disorders |  |  | F20.0-F20.3, F20.5, F20.81, F20.89, F20.9, F25.0-F25.1, F25.8-F25.9 |
| Schizophrenia & Related Disorders (ICD-09) |  |  | 297.0-297.3, 297.8-297.9, 298.0-298.4, 298.8-298.9 |
| **MH utilization** |  |  |  |
| HCPCS - Behavioral Health Services |  | G0410-G0411G2214, T1006 |  |
| H Codes - Community & Mental Health Services |  | H0004-H0005, H0050, H2017-H2020, H2033 |  |
| Psychiatric Diagnostic & Evaluation Services |  | 90785, 90791-90792 |  |
| Psychotherapy Services |  | 90832-90834, 90836-90838 |  |
| Crisis Psychotherapy & Other Psych Services |  | 90839, 90840, 90845-90847, 90849, 90853 |  |
| Other Psychiatric & Psychotherapy Services |  | 90870, 90875, 90876, 90880, 90889 |  |
| Neuropsychological & Psychological Testing |  | 96105, 96112, 96113, 96116, 96121, 96130, 96131-96133, 96136-96139, 96146 |  |
| Health & Behavior Assessment/Intervention |  | 96152–96156, 96158, 96159, 96161, 96164 |  |
| Behavioral Health Care Coordination |  | 99484 |  |
