# Supplementary material for: Mental Health and Survival in Medicare Beneficiaries With Lung and Head and Neck Cancer
Source: Psychooncology. 2026 Jun 4;35(6):e70510. doi: 10.1002/pon.70510 (PMC13238330; doi:10.1002/pon.70510)
Supplement: Supplementary file 2 — Table S2: Cox model and cancer specific survival hazard of death among lung (model 1) and head and neck (HN) (model 2) cancers with a mental health diagnosis and with and without mental health service. [file PON-35-e70510-s002.docx]

Supplemental table 2. Cox model and cancer specific survival hazard of death among lung (model 1) and head and neck (HN) (model 2) cancers with a mental health diagnosis and with and without mental health service.

|  | Model 1 (Lung) | Model 2 (HN) |
| --- | --- | --- |
|  | Hazard ratio (95% CI) | Hazard ratio (95% CI) |
|  |  |  |
| Age |  |  |
| 67-75 | 1.00 Referent | |
| 75+ | 1.14 (1.12-1.17) | 1.17 (1.06-1.29) |
| Sex |  |  |
| Male | 1.00 Referent | |
| Female | 0.80 (0.77-0.82) | 0.82 (0.74-0.91) |
| Marriage status | |  |
| Yes | 1.00 Referent | |
| No | 1.16 (1.12-1.18) | 1.19 (1.07-1.32) |
| Missing | 0.10 (0.10-0.11) | 0.85 (0.80-0.91) |
| Race |  |  |
| NHW | 1.00 Referent | |
| Black | 1.05 (1.01-1.10) | 1.25 (1.03-1.53) |
| Other | 0.91 (0.85-0.99) | 1.04 (0.80-1.34) |
| Urban/Rural | |  |
| Urban | 1.00 Referent | |
| Rural | 1.10 (1.06-1.15) | 0.94 (0.79-1.12) |
| Missing | 1.08 (0.97-1.20) | 0.88 (0.51-1.53) |
| Stage |  |  |
| I-II | 1.00 Referent | |
| III | 1.45 (1.31-1.60) | 1.06 (0.80-1.40) |
| IV | 3.55 (3.45-3.65) | 2.04 (1.83-2.28) |
| Missing | 1.69 (1.60-1.79) | 1.63 (1.37-1.95) |
| Charlson Index |  |  |
| 0 | 1.00 Referent | |
| 1 | 1.02 (0.99-1.05) | 1.17 (1.03-1.34) |
| 2+ | 1.05 (1.02-1.08) | 1.34 (1.20-1.50) |
| Mental health services |  |  |
| No | 1.00 Referent | |
| Yes | 0.65 (0.62-0.67) | 0.59 (0.51-0.68) |
